# Application of Deep Eutectic Solvents for the Extraction of Rutin and Rosmarinic Acid from *Satureja montana* L. and Evaluation of the Extracts Antiradical Activity

**DOI:** 10.3390/plants9020153

**Published:** 2020-01-26

**Authors:** Martina Jakovljević, Jelena Vladić, Senka Vidović, Kristian Pastor, Stela Jokić, Maja Molnar, Igor Jerković

**Affiliations:** 1 Faculty of Food Technology Osijek, Josip Juraj Strossmayer University of Osijek, Franje Kuhača 20, 31000 Osijek, Croatia; mjakovljevic@ptfos.hr (M.J.); sjokic@ptfos.hr (S.J.); mmolnar@ptfos.hr (M.M.); 2 Faculty of Technology, University of Novi Sad, Bulevar cara Lazara 1, 21000 Novi Sad, Serbia; vladicjelena@gmail.com (J.V.); senka.vidovic@uns.ac.rs (S.V.); herr.pastor.kristian@gmail.com (K.P.); 3 Faculty of Chemistry and Technology, University of Split, Ruđera Boškovića 35, 21000 Split, Croatia

**Keywords:** deep eutectic solvents, extraction, rutin, rosmarinic acid, *Satureja montana* L., principal component analysis

## Abstract

*Satureja montana* L. was used in the current research as the plant exhibits numerous health-promoting benefits due to its specific chemical composition. The extraction method based on deep eutectic solvents (DESs) was used for the extraction of rutin and rosmarinic acid from this plant. Five different choline chloride-based DESs with different volumes of water (10%, 30%, and 50% (*v*/*v*)) were used for the extraction at different temperatures (30, 50, and 70 °C) to investigate the influence on rosmarinic acid and rutin content obtained by high-performance liquid chromatography with diode-array detector (HPLC-DAD) in the obtained extracts. A principal component analysis was employed to explore and visualize the influence of applied parameters on the efficiency of the extraction procedure of rutin and rosmarinic acid. Among the tested DESs, choline chloride:lactic acid (mole ratio 1:2) and choline chloride:levulinic acid (mole ratio 1:2) were the most suitable for the extraction of rutin, while for rosmarinic acid choline chloride:urea (mole ratio 1:2) was the most effective solvent. The extract showing the best antiradical activity was obtained with choline chloride:urea (mole ratio 1:1) at 30 °C and 50% H_2_O (*v/v*).

## 1. Introduction

In recent decades, the growth of the pharmaceutical industry, particularly in the field of products rich in bioactive components for maintaining health, has led to the rapid development of extraction and isolation methods [1]. This development is directed towards modern extraction methods, which are not only faster but also more efficient, providing greater yields and better quality of the extracts without the use of flammable and toxic organic solvents [2].

Among these extraction methods, deep eutectic solvents (DESs) were presented by Abbott et al. [3,4] as a continuation of ionic liquids, despite a significant difference in starting material and in the way they are synthesized with respect to ionic liquids. DESs are a mix between the hydrogen bond acceptor (HBA) and a hydrogen bond donor (HBD) with a lower melting point relative to the melting point of the individual starting components. DESs have been popular in recent years due to the low price of starting materials and easy preparation of the solvent, as well as biodegradability, low toxicity [5], and tunable properties [6,7,8]. They have been used in various fields, including biotechnology and the chemical industry, since they present promising solvents for extraction and separation processes. The application of DES for extraction and isolation has been successfully applied in several fields, such as biodiesel, bioactive components, metals, and aromatic hydrocarbons [1].

Since there are numerous different combinations of starting materials and mole ratios, DESs can actually be considered as tunable solvents with different functionality and solubility for various compounds. In addition, this brings the possibility of increasing the solubility and extraction efficiency of DESs by selecting the appropriate combination of HBD and HBA, as well as their mole ratios. Choline chloride, as one of the commonly used compounds as HBA (acceptable price, non-toxic influence, and the possibility of biodegradation), can form DESs with a range of different components, including carboxylic acids, sugars, sugar alcohols, and amines as HBDs [3,4]. During the last years, DESs have become extensively used as solvents for the extraction of phenolic compounds, such as phenolic acids, anthocyanins, flavonoids, furanocoumarins, and stilbenes [9,10,11,12].

*Satureja montana* L. has long been present in traditional medicine. Its common use as a traditional remedy in the therapy of different digestive, respiratory, and urinary ailments represented the basis and reason behind conducting numerous scientific studies on this plant. Those studies confirmed numerous biological activities of *S. montana,* such as cardioprotective (through angiotensin-converting enzyme (ACE) inhibition), diuretic, antimicrobial, antidiarrheal, hepatoprotective, cytotoxic, and antioxidative [13,14,15,16,17,18,19,20,21]. Additionally, *S. montana* could be considered for the treatment of premature ejaculation [22]. Carvacrol and thymol are the most dominant components of its essential oil. A large number of published studies focused on the investigation of its essential oil and lipophilic components. The most applied methods for obtaining volatile compounds of *S. montana* were hydro distillation and Soxhlet extraction [23,24,25,26,27,28,29]. However, with the development of green modern extraction technologies, which overcome the shortcomings of classical extraction techniques, studies were conducted to obtain lipophilic extracts of *S. montana* by applying green extraction methods, such as supercritical carbon dioxide extraction [25,27,30,31], microwave-assisted hydro distillation [32], and subcritical water extraction [20]. Apart from lipophilic extracts, *S. montana* represents a source of hydro soluble antioxidants, such as rutin, quercetin, caffeic, rosmarinic, *p*-coumaric, ellagic, protocatehuic, rosmarinic, and syringic acid [21,27,33,34].

Rutin (30,40,5,7-tetrahydroxy-flavone-3-rutinoside), flavonol glycoside, is a bioactive compound with reported clinically relevant activities, such as antioxidant, anti-inflammatory, antimicrobial, anti-tumor, and anti-asthma [35]. Today, it is found in more than 70 plant species, and given its many health effects, it is a component that is often extracted while trying to find optimal extraction conditions. Extraction methods for rutin include a wide range of different techniques such as the Soxhlet extraction [35], ultrasound-assisted extraction [36], microwave-assisted extraction [37], infrared assisted solvent extraction [38], pressurized liquid extraction [39], the mechanochemical method [40], and supercritical fluids [41], as well as deep eutectic solvents [42,43]. On the other hand, rosmarinic acid, which is known as labiatenic acid and consists of an ester of caffeic acid and 3,4-dihydroxyphenyllactic acid, is a phytochemical with numerous pharmacological activities including photoprotective, anticancer, antidepressive agent, as well as agent in inhibition of angiogenesis and in the prevention of neurodegenerative disease [44]. Because of its attractiveness, rosmarinic acid is extracted by a variety of techniques including conventional (maceration, heat reflux, Soxhlet extraction) and innovative extraction techniques (ultrasound- and microwave-assisted extraction, supercritical fluid extraction, pressurized liquid extraction) [45].

Therefore, *S. montana* deserves increased scientific attention due to its chemical composition and health-promoting benefits. Moreover, there is a constant demand for the development of new products based on natural materials through the application of new, convenient, rapid, and eco-friendly technologies.

Considering all the aforementioned advantages of the application of DESs and their determined extraction potential on different samples, the objectives of this study were focused on the possibilities of the application of these green and sustainable solvents for the extraction of targeted bioactive components (rutin and rosmarinic acid) from *S. montana.* The influence of parameters (temperature and water content) on the number of bioactive components in the extracts and their antiradical activity was investigated. Principal component analysis (PCA) was utilized to reveal the relationships between the extraction parameters and extraction efficiency of targeted compounds. A literature search showed that there is no available data on the extraction with DESs and its optimization for rosmarinic acid and rutin extraction from *S. montana*. To the authors’ best knowledge, this is the first study of DESs extraction of the bioactive components from *S. montana* and the antiradical activity of these extracts. The antiradical activity of the extracts was determined by a DPPH (2,2-diphenyl-1-picrylhydrazyl) assay as it represents the most frequently employed method for the determination of antiradical capacity. The reason for its wide application lies in its simplicity and efficiency in terms of cost and time [46]. Furthermore, given its application and frequency of use, it is easier to compare the obtained results with those obtained in other studies.

## 2. Results

Given that DESs are different (different components are used as HBDs which affect the physicochemical properties of the solvent itself) as well as their ability to dissolve and extract different components, it is hard to estimate the suitability of DESs for the extraction of the highest amount of desired components. That is the reason why, in this research, five different DESs and different extraction parameters were evaluated for the extraction of targeted bioactive components from *S. montana*.

PCA is used as a tool able to provide an overview of the interrelationships that exist in the data sets. This method is generally used for revealing relations between variables and between samples (e.g., patterns), detecting outliers, finding and quantifying patterns and trends, extracting and compressing multivariate data sets, among other applications [47].

### 2.1. Comparison of the Possibility of Extraction by DESs

**Figure 1:**
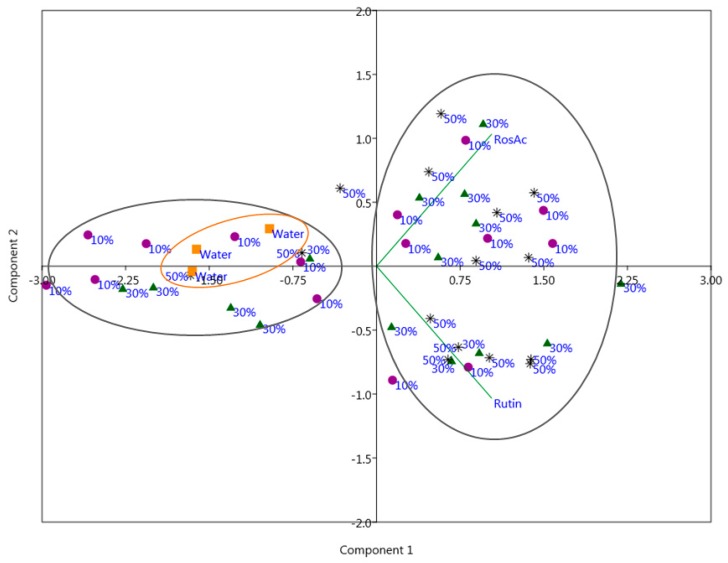
Principal component analysis (PCA) biplot of the extracts with scores representing various H_2_O content (10%, 30%, and 50% (*v*/*v*)) and pure water utilized in the extraction procedure.

**Figure 2:**
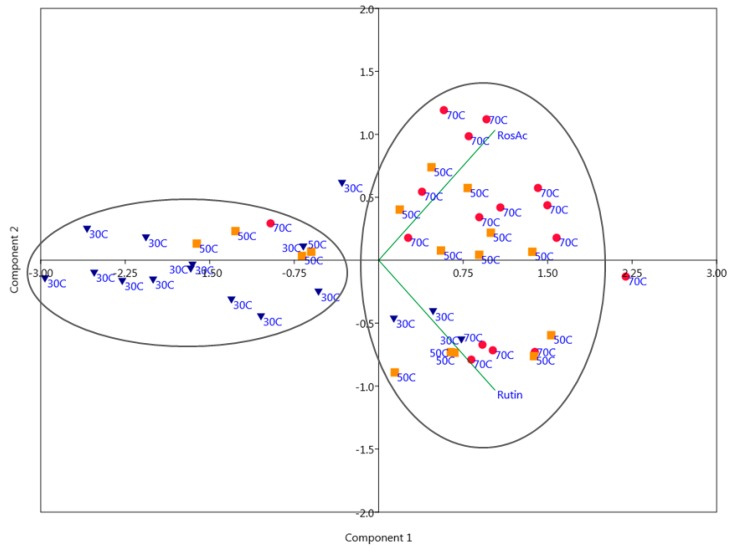
Biplot of the extracts with scores representing various temperatures (30, 50, and 70 °C) utilized in the extraction procedure.

**Figure 3:**
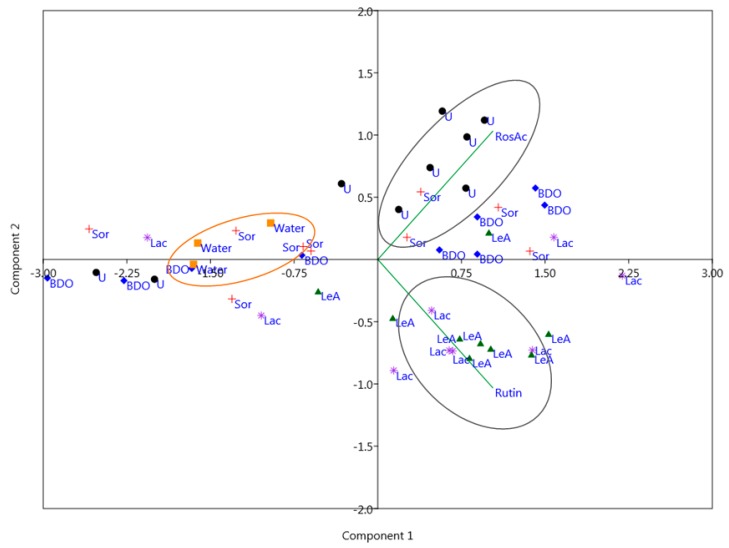
PCA biplot of the extracts with scores representing various solvent types (ChCl-U, ChCl-Sor, ChCl-BDO, ChCl-Lac, ChCl-LeA, and pure H_2_O) utilized in the extraction procedure.

As can be seen from Table S1 and Figure 1, Figure 2 and Figure 3, DESs differ in their ability to extract rutin and rosmarinic acid from *S. montana*. The results showed that not only the type of HBD affected the extraction efficiency but also the extraction parameters. In this study, the amount of H_2_O added and temperature were varied parameters since they affect the physicochemical properties of DESs and also influence the amount of extracted compounds [48]. The viscosity of DESs represents one of the biggest drawbacks due to a slow mass transfer, but viscosity can be reduced just by adding H_2_O or increasing the extraction temperature [49]. Apart from reducing viscosity, the addition of water and an increase in temperature can affect pH [50], enabling the extraction of compounds over a wider pH range.

Although the addition of H_2_O can change the physicochemical properties of DESs, making them even more tunable solvents, an excessive amount of H_2_O can decrease the interaction between components of DESs, as well as the interaction between DESs and the extracted components, [9] reducing their extraction efficiency. Because of all the aforementioned, the range of temperature and H_2_O addition were investigated to make an adequate conclusion about the parameters required for efficient extraction.

Five chosen DESs showed the ability to extract rutin and rosmarinic acid. Although it is possible to extract rosmarinic acid and rutin with all of the applied DESs, ChCL-Lac (choline chloride:lactic acid (mole ratio 1:2)) and ChCL-LeA (choline chloride:levulinic acid (mole ratio 1:2)) were the most suitable for the extraction of rutin while for rosmarinic acid similar amount was obtained with all tested DESs although ChCl-U (choline chloride:urea (mole ratio 1:2)) stood out as the most effective solvent. It is noticeable from Figure 1, Figure 2 and Figure 3 that the amount of rutin depended on temperature and H_2_O addition as well as on DES used since the highest amount of rutin was not obtained in all DESs under the same extraction parameters. In most cases, an increased amount of rutin was achieved at higher temperatures with more added H_2_O, probably due to lower viscosity and faster mass transfer. Since higher H_2_O content contributes to better extraction efficiency, the extraction with H_2_O was carried out in the same manner, during 60 min and at the same temperatures as in DESs extraction. In the extraction with H_2_O as a solvent, 4.75 ± 0.28–6.72 ± 0.29 µg mg^−1^ of rutin and 2.30 ± 0.56–3.81 ± 0.54 µg mg^−1^ of rosmarinic acid were extracted, depending on applied temperature. According to the results, the mixture of DESs and H_2_O in a different ratio, especially 30% and 50% (*v*/*v*), is an excellent option for the extraction of rutin and rosmarinic acid from *S. montana*.

### 2.2. Principal Component Analysis

Figure 1 shows the PCA biplot of the extracts with scores representing various solvent shares used for the extraction process of rutin and rosmarinic acid. Both rutin and rosmarinic acid correlate significantly with PC1 in a positive manner while less significantly with PC2—rosmarinic acid in positive, and rutin in a negative manner. The scores of extracts with H_2_O amount of 30% and 50% (*v*/*v*) (represented as green triangles and black stars) are mainly positioned on the right side of the PCA diagram, thus also exhibiting positive correlations with PC1. On the other hand, the extracts with the lower H_2_O amount of 10% (*v*/*v*) (purple dots) and pure H_2_O (orange squares) are positioned on the left side of the PCA diagram, indicating a negative correlation with both rutin and rosmarinic acid. Figure 1 suggests that increasing H_2_O content in the extraction procedure improves the extraction efficiency while decreasing H_2_O content or using pure H_2_O showed a negative impact on the extraction procedure.

Figure 2 shows the PCA biplot of the extracts with scores representing various temperatures used for the extraction process of rutin and rosmarinic acid. The extracts obtained under higher temperatures (50 and 70 °C; orange squares and red dots) correlate in a positive manner with PC1, along with the extracted components—rutin and rosmarinic acid. Thus, these points are located on the right side of the PCA diagram, close to the extracted components. The extracts obtained using lower temperatures (10 °C; blue triangles) correlate negatively with PC1 and are thus located on the left side of the PCA diagram. Figure 2 indicates that higher temperatures used in the extraction procedure can positively influence the extraction of both rutin and rosmarinic acid from investigated plant material.

Figure 3. shows the PCA biplot of the extract samples with scores representing various solvent types (ChCl-U, ChCl-Sor, ChCl-BDO, ChCl-Lac, ChCl-LeA, and pure H_2_O) utilized in the extraction procedure. Most scores of the extracts obtained using solvents ChCl-U (represented as black dots) and few scores of ChCl-Sor (red plus) are located close to the rosmarinic acid variable, and almost all scores of the extracts obtained using ChCL-LeA (green triangles) and ChCl-Lac (purple stars) are located close to the rutin variable, thus suggesting that these solvents stimulate the extraction of components they correlate intensively with. However, the majority of scores of the extracts obtained using ChCl-BDO (blue diamonds) do not have a strong correlation with any of the extracted compounds. Scores of the extracts obtained using pure H_2_O (orange squares) correlate negatively with PC1 and are thus located on the left side of the PCA diagram, suggesting that pure H2O could not be used to efficiently extract rutin and rosmarinic acid from analyzed plant material under the conditions used.

### 2.3. Antioxidant Activity

**Figure 4:**
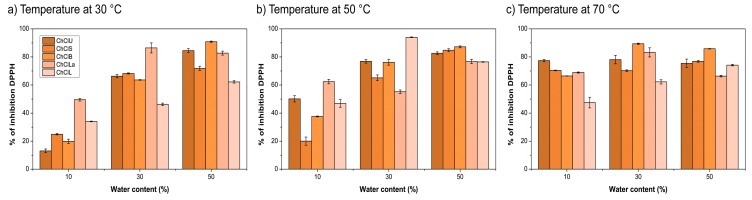
The antiradical activity of the samples shown as percentage inhibition of DPPH (**a**-temperature at 30 °C; **b** a-temperature at 50 °C; **c**-temperature at 70 °C).

DPPH inhibition in percent for all the samples concentrations of 1 mgmL^−1^ is presented in Figure 4, ranging from 13.07% ± 1.35%–94.00% ± 0.25 %.

For each DES, the sample showing the highest percentage of DPPH inhibition was selected, and EC_50_ was determined (Table 1). According to EC_50_, the solvents with provided the extracts with the best antiradical activity were ChCl-U and ChCl-La at 30 °C and with the addition of 50% and 30% H_2_O (*v*/*v*), respectively.

## 3. Discussion

### 3.1. Comparison of the Possibility of Extraction by DESs

According to the literature [42,43], rutin is extracted from different plants with DESs, especially with acidic DESs, such as choline chloride:citric acid (mole ratio 2:1) or proline:2,3-diaminosuccinic acid, which is in agreement with our results. For the analysis of rutin, which is one of the frequently extracted components using DESs, the COSMO-RS program (COSMOConfX16software; SCM Software for Chemistry and Materials, Amsterdam, The Netherlands) was used to analyze 126 DESs, showing that the most effective solvents were cyclic. According to this research, the most adequate for the rutin extraction was the use of carboxylic acids with two carboxyl groups and a main chain consisting of two methylene groups with two amino substituents. Since rutin has acidic properties, the presence of basic sites on the components of the DESs could be the reason for the improved extraction. In the case of the preparation of DESs with carboxylic acids as HBDs for extraction of rutin, the high acidity of the carboxylic acid component of the DESs and a large number of highly basic centers is important, which was also confirmed by our results [43].

The quantities of extracted rutin ranged from 1.40 ± 0.03 to 17.29 ± 0.64 µg mg^−1^ of the plant, depending on applied parameters and DESs. The amount was much higher in comparison with the results obtained in the paper by Kremer at al. [51], where the extracts were prepared by ultrasonication of powdered material with 80% ethanol and methanol. In methanolic and ethanolic extracts of *S. montana* the amount of rutin was 0.07%–0.15%, while in current research, the amount was 0.14%–1.67%, depending on the applied parameters and solvents. In our study, by comparing the results of classical solvents and DESs, we also determined that a smaller amount of rutin was extracted with classical solvents. The amounts of extracted rutin ranged from 1.18 to 10.45 µg mg^−1^ of the plant, depending on the parameters applied and solvent, which is less compared with DESs. The highest amount of rutin was extracted with 50% ethanol at 70 °C (Table S1). An increasing number of studies have shown that DESs are more suitable solvents for the extraction of rutin with much greater extraction efficiency in comparison to H_2_O and organic solvents [46].

In the case of rosmarinic acid, only a few papers focused on extraction using different solvents, showing that the extraction capacity of ChCl-based DESs was markedly higher than that of Bet- and Pro-based DESs. In addition, amides-based DES showed the highest efficiency compared to the other three types of HBDs. This part has been added to the manuscript.

In the case of rosmarinic acid, the influence of parameters was different. An increased amount of rosmarinic acid was obtained with a higher addition of H_2_O, but increased temperature showed an important influence only in the experiment with 10% of H_2_O (*v*/*v*) probably due to lower viscosity at higher temperatures. This was also determined with alcohol-based deep eutectic solvents, where 40% of H_2_O provided the highest yield of rosmarinic acid, but the yield was higher at higher temperatures in all experiments [52]. In a paper by Duan et al. [53], DESs with different HBAs were prepared for polyphenol extraction, and ChCl-based DESs proved to be more suitable for rosmarinic acid extraction than Bet- and Pro-based DESs. In addition, the highest extraction capacity is shown by amides-based DES relative to other HBDs, which is consistent with our results.

The quantities of extracted rosmarinic acid ranged from 0.21 ± 0.01 to 7.85 ± 0.32 µg mg^−1^ of the plant, depending on applied parameters and solvents. Unlike the amount of rutin, the amount of extracted rosmarinic acid was lower than in prepared methanol and ethanol extracts by Kremer at al. [51]. In Reference [51], the obtained amount of rosmarinic acid was 0.77%–1.44% and 1.11%–1.58% in methanolic and ethanolic extracts, respectively, while in the present study, it was 0.02%–0.78%. In the paper by El Tawab et al. [33], rutin and rosmarinic acid were also the main components but in smaller quantities compared to present results (the amount of rosmarinic acid was about 0.365 µg mg^−1^ while rutin concentration was about 1.135 µg mg^−1^). According to our results, the amount of rosmarinic acid extracted with conventional solvents was 0.33 ± 0.11–7.44 ± 0.03 µg mg^−1^, which is similar to the results obtained using DESs. The highest amount of rosemary acid was extracted using 50% ethanol at 70 °C (Table S1).

### 3.2. Antiradical Activity

DPPH assay, which is one of the most commonly used methods, was used to determine antiradical activity (DPPH inhibition in percentage ranged from 13.07% to 94.00%). With the increase in H_2_O content, percentage DPPH increases, but with the increase in temperature, the increase in percentage DPPH is observed only in cases where the H_2_O content is lower. This may be due to a decrease in viscosity at higher temperatures and, thus a better mass transfer and more efficient extraction of phenolic components.

The powerful antioxidant capacity of both components was demonstrated by different antioxidant assays [35,44,45], meaning that both components, as well as other unexplored components in the sample, influence the DPPH assay results. This can also be seen by the correlation between these components and antiradical activity, which for rutin and rosmarinic acid were 0.54 and 0.73, respectively. From this, it can be observed that rosmarinic acid has a greater effect on antiradical activity and other components, and their synergism potentially affects the activity of the extract as well.

Since EC_50_ was obtained for the samples showing the highest percentage of DPPH inhibition, it is possible to compare the obtained results with the literature data. According to EC_50_, the sample having the best antiradical activity of 100.64 ± 15.74 µg mL^−1^ can be compared with methanolic extract of *S. montana* where EC_50_ was 116.36 ± 12.83 µg mL^−1^ [54].

## 4. Materials and Methods

*Satureja montana* L. was collected at the Institute of Field and Vegetable Crops, Backi Petrovac, Republic of Serbia. The collected plant material was air-dried, milled, and mean particle size (0.301 mm) was determined by sieves set (Erweka, Germany).

### 4.1. Preparation of DESs

The choline chloride-based DESs were prepared, as described in our previous paper [42]. In this study, five different DESs were prepared using available and inexpensive components, as shown in Table 2.

### 4.2. DESs Extraction of Bioactive Components

Ground *S. montana* L. dried leaves (50 mg) were mixed with 1 mL of selected solvent which was a mixture of DESs and ultrapure H_2_O (Millipore Simplicity 185, Darmstadt, Germany). DESs and ultrapure water were mixed in different proportions (10%, 30%, 50% of water (*v/v*)) to reduce the viscosity of the solvent itself, and the solvents prepared in this way were used to extract *S. montana* dried leaves. To compare the results, extracts were also obtained with conventional solvents (30%, 50%, and 70% ethanol (*v/v*) and methanol) in the same manner.

Prepared samples were stirred at 1500 rpm in an aluminum block (Stuart SHB) on a magnetic stirrer at a specified temperature (30, 50, or 70 °C) for 60 min. After the extraction, the mixture was centrifuged for 15 min and then decanted. The supernatant liquid was diluted with methanol, filtered through a PTFE 0.45 μm filter, and subjected to HPLC analysis.

### 4.3. Chemical Characterization of the Extracts

HPLC analysis was performed on an Agilent 1260 Infinity II (Analytical Instruments, CA, USA), and chromatographic separation was obtained on a ZORBAX Eclipse Plus C18 (Agilent, Santa Clara, United States) column (100 × 4.6 mm, 5 µm). The separation of analyzed compounds was performed with gradient elution for 47 minutes where acetonitrile was used as phase B and 0.1% CH_3_COOH (in Millipore water) as phase A, in accordance with the following profile: 2%–4% B (10 min), 4%–5% B (5 min), 5% B (5 min), 5%–45% B (17 min), and 45% to 2% B (10 min). The flow rate was 1.0 mL/min, the injection volume was 20 μL, and the UV detection wavelength was 260 and 330 nm. The chromatography was performed at room temperature (22 °C). Standard stock solutions for rutin and rosmarinic acid were prepared in a solvent, and calibration was obtained at eight concentrations (concentration range 20.0, 30.00, 50.0, 75.0, 100.0, 150.00, 200.0, mg L^−1^). The linearity of the calibration curve was confirmed by R^2^ = 0. 99537 for rutin and R^2^ = 0.99856 for rosmarinic acid.

### 4.4. Antiradical Activity

The DPPH (2,2-diphenyl-1-picrylhydrazyl) method was performed to determine the antiradical activity of the extracts according to the method previously described [55]. Methanol solution of DPPH (0.3 mM) was prepared daily and kept in a dark place. Before the measurements, the absorbance of the DPPH solution was determined at the same conditions. Prepared solutions of the extracts at a concentration of 1 mg mL^−1^ (1.2 mL) with added DPPH solution (0.5 mL) were stored in the dark for 30 min. The absorbance was determined after 30 min at 517 nm using a spectrophotometer (Helios γ; Thermo Spectronic, Cambridge, Great Britain). All measurements were done in triplicate, compared with control blank and the DPPH activity was calculated using the following equation:

DPPH activity (%) = (((A_DPPH_+ A_S_) - A_S_)/A_DPPH_) × 100 (1)

For the samples showing a significantly higher inhibition, EC_50_ was determined to compare the results with the literature data where different methods and solvents were used [54]. EC_50_ represents one of the most commonly determined parameters by DPPH assay for the determination of not only the antiradical capacity but also for the comparison of the activities of different components or extracts, and it refers to the concentration of extract required to reduce the absorbance of DPPH by 50% [56].

The samples obtained by five different DESs, showing the best percentage inhibition of DPPH radical prepared in different concentrations, were used for calculating EC_50_ values by preparing the curves from the obtained relative scavenging capacity values.

### 4.5. Data Processing

The HPLC results were further subjected to principal component analysis (PCA) to reveal the correlations between considered extract samples and variables in the data sets [57]. In general, the main objectives of multivariate methods, and thus PCA as an essential one, include data reduction, grouping and the classification of observations, and the modeling of relationships that may exist between variables [58]. In this specific case, PCA was utilized to investigate the potential influence of employed parameters on the efficiency of a green extraction procedure in obtaining bioactive compounds—rutin and rosmarinic acid (RosAc)—from *S. montana* plant material. Simultaneous comparison of the score and loading plots gives an insight into the relationship between samples—obtained extracts, and variables observed—extracted bioactive compounds. The obtained correlation matrix PCA biplots showed both loadings (rutin and rosmarinic acid) and scores (employed extraction parameters: water addition, temperature, and solvent type used) divided into three separate PCA diagrams, thus enabling the observation of the influence of every parameter individually. Considering the bivariate nature of the input data, the first two principal components, constituting a two-dimensional space (PC1 vs. PC2), explained a total variance of analyzed input data—100%. All calculations were performed using freely available PAST 3.15 software [59].

### 4.6. Statistical Analysis

The experiments were carried out in triplicate, and the results were expressed as mean value ± standard deviation and considered significantly different when *p* ≤ 0.05. One-way ANOVA was conducted to test the influence of individual factors on the observed property, and Tukey’s HSD post hoc test was used to determine the differences between the mean values (STATISTICA v. 13 free trial).

## 5. Conclusions

In this study, the determination of suitable DES for the extraction of rutin and rosmarinic acid from *S. montana* was investigated. The obtained amount of rutin was 1.40 to 17.29 μg mg^−1^ of the plant and of rosmarinic acid 0.21 to 7.84 μg mg^–1^, depending on the applied parameters and solvents. Among five different solvents, choline chloride:lactic acid (mole ratio 1:2) and choline chloride:levulinic acid (mole ratio 1:2) were the most appropriate for the extraction of rutin while for rosmarinic acid choline chloride:urea (mol ratio 1:2) was the most suitable solvent. The results of PCA suggest that increasing the extraction temperature and decreasing the H_2_O amount can increase the extraction of bioactive components. Furthermore, ChCl-U solvent demonstrated to be the most efficient in the extraction of rosmarinic acid, while the solvents ChCl-LeA and ChCl-Lac stimulate the extraction of rutin from *S. montana*. The percentage of DPPH inhibition in all samples ranged from 13.07 to 94.0, and the sample with the best antioxidant activity (EC_50_ = 100.64 ± 15.74 µg mL^−1^) was the one with choline chloride:urea (mol ratio 1:2) made at 30 °C with 50% H_2_O addition (*v*/*v*). Compared to the extracts obtained with organic solvents, the amounts of extracted rutin and antiradical activity are higher in the extracts obtained using DESs, while the amount of rosmarinic acid is lower than in the extracts obtained with organic solvents. DESs are a greener alternative to organic solvents, and because of their design capabilities and tunable properties, they can be customized further for the extraction of desired components as well as for use in various sectors, such as pharmaceutical, cosmetic and food industry. Given that the area of DESs application is relatively new, further research is needed to find the optimal solvent for certain components as well as for groups of bioactive components.

## Figures and Tables

**Table 1:** EC_50_ values of extracts with the highest percentage inhibition of DPPH.

Solvent	Parameters	EC_50_ (µg mL^−1^)
ChCl-U	30 °C 50% H_2_O	100.64 ± 15.74
ChCl-Sor	50 °C 50% H_2_O	568.05 ± 21.51
ChCl-BDO	30 °C 50% H_2_O	497.96 ± 2.82
ChCL-Lac	30 °C 30% H_2_O	207.03 ± 6.76
ChCL-LeA	50 °C 30% H_2_O	459.01 ± 8.83

**Table 2:** List of prepared deep eutectic solvents (DESs) for the extraction.

	Components		Mole Ration (HBA:HBD)	Appearance
	Hydrogen Bond Acceptor (HBA)	Hydrogen Bond Donors (HBDs)		
ChCl-U	Choline chloride	Urea	1:2	Clear and in liquid state at 80 °C
ChCl-Sor		Sorbitol	1:1	Clear, viscous and in liquid state at 80 °C
ChCl-BDO		Butane-1,4-diol	1:2	Clear and in liquid state at 80 °C
ChCL-Lac		Lactic acid	1:2	Clear and in liquid state at 80 °C
ChCL-LeA		Levulinic acid	1:2	Clear and in liquid state at 80 °C

## Supplementary Materials

The following are available online at https://www.mdpi.com/2223-7747/9/2/153/s1, Table S1. Content of rutin and rosmarinic acid in extracts obtained with different solvents and under different conditions.
