# Supplementary material for: Application of Deep Eutectic Solvents for the Extraction of Rutin and Rosmarinic Acid from Satureja montana L. and Evaluation of the Extracts Antiradical Activity
Source: Plants (Basel). 2020 Jan 26;9(2):153. doi: 10.3390/plants9020153 (PMC7076517; doi:10.3390/plants9020153)
Supplement: Supplementary file 1 [file plants-09-00153-s001.pdf]

Table S1. Content of rutin and rosmarinic acid in extracts obtained with different solvents and under different conditions

Type of solvent	Extraction parameters		Rutin (µg/mg)	Rosmarinic Acid (µg/mg)	DPPH Inhibition (%)
	Water Content (%)	Temperature of Extraction (°C)			
**ChCl-U**	**10**	**30**	2.67 ± 0.70 ^{BCD}	0.92 ± 0.05 ^{wxy}	13.07 ± 1.35 ^{w}
		50	9.47 ± 0.57 $^{m-r}$	5.66 ± 0.61 $^{c-j}$	66.30 ± 1.22 $^{m-p}$
		70	9.56 ± 0.92 $^{m-q}$	7.42 ± 1.20 ^{ab}	84.49 ± 1.36 ^{f}
	30	30	4.45 ± 1.03^{y-B}	1.61 ± 0.45 $^{t-y}$	50.15 ± 2.20 ^{r}
		50	10.80 ± 0.69^{i-n}	6.80 ± 0.09^{a-d}	76.92 ± 1.34^{hi}
		70	9.63 ± 0.16^{l-q}	7.85 ± 0.32^{a}	82.61 ± 1.02^{fg}
	50	30	7.25 ± 0.76^{r-w}	5.21 ± 0.57^{e-l}	77.40 ± 0.86^{hi}
		50	9.30 ± 1.22^{n-s}	6.57 ± 0.11^{a-f}	78.09 ± 3.02^{gh}
		70	8.24 ± 0.37^{o-u}	7.40 ± 0.11^{ab}	75.48 ± 3.02^{hij}
ChCl-Sor	10	30	1.40 ± 0.03^{CD}	1.34 ± 0.01^{t-y}	24.92 ± 0.43^{u}
		50	6.01 ± 1.04^{u-y}	3.26 ± 0.58^{n-s}	68.24 ± 0.43^{l-o}
		70	10.39 ± 0.50^{j-p}	5.44 ± 0.78^{d-k}	71.83 ± 1.55^{jkl}
	30	30	7.08 ± 0.06^{s-w}	2.40 ± 0.35^{q-v}	20.03 ± 2.90^{v}
		50	8.08 ± 0.16^{q-u}	4.01 ± 0.02^{k-p}	65.13 ± 2.04^{nop}
		70	9.64 ± 0.12^{l-q}	6.16 ± 0.11^{b-g}	84.79 ± 1.02^{ef}
	50	30	7.75 ± 0.00^{q-v}	3.96 ± 0.34^{l-p}	70.39 ± 0.25^{klm}
		50	14.13 ± 0.66^{c-g}	6.90 ± 0.26^{abc}	70.22 ± 0.55^{klm}
		70	12.17 ± 0.19^{f-k}	6.88 ± 0.17^{abc}	76.85 ± 0.61^{hi}
ChCl-BDO	10	30	1.46 ± 0.20^{CD}	0.21 ± 0.01^{y}	19.90 ± 1.52^{v}
		50	7.93 ± 1.43^{q-v}	3.84 ± 0.75^{l-p}	63.62 ± 0.20^{op}
		70	13.40 ± 0.31^{c-h}	7.64 ± 0.21^{a}	90.85 ± 0.46^{ab}
	30	30	3.64 ± 0.04^{z-C}	1.19 ± 0.14^{u-y}	37.59 ± 0.30^{t}
		50	11.60 ± 0.07^{h-m}	5.72 ± 0.09^{c-i}	37.59 ± 2.06^{t}
		70	11.83 ± 0.36^{h-l}	6.61 ± 0.05^{a-e}	87.20 ± 0.67^{b-f}

	50	30	5.21 ± 0.15$^{\text{w-A}}$	2.23 ± 0.05$^{\text{s-w}}$	66.47 ± 0.12$^{\text{m-p}}$
		50	12.75 ± 0.72$^{\text{e-i}}$	6.17 ± 0.29$^{\text{b-g}}$	89.41 ± 0.42$^{\text{a-e}}$
		70	12.72 ± 0.51$^{\text{e-i}}$	7.72 ± 0.06^{a}	85.83 ± 0.17$^{\text{c-f}}$
ChCL-Lac	10	30	3.22 ± 0.19$^{\text{A-D}}$	1.94 ± 0.10$^{\text{s-x}}$	49.51 ± 0.91^{r}
		50	13.32 ± 0.13$^{\text{c-h}}$	3.69 ± 1.19$^{\text{m-r}}$	86.43 ± 3.57$^{\text{b-f}}$
		70	14.45 ± 1.17$^{\text{b-e}}$	7.38 ± 0.57^{ab}	82.75 ± 1.43^{fg}
	30	30	8.30 ± 1.33$^{\text{o-t}}$	2.59 ± 0.59$^{\text{p-u}}$	62.41 ± 1.58^{p}
		50	14.47 ± 1.20$^{\text{b-e}}$	4.70 ± 0.53$^{\text{h-m}}$	55.21 ± 1.27^{q}
		70	17.29 ± 0.64^{a}	7.83 ± 0.02^{a}	76.75 ± 1.55^{hi}
	50	30	12.88 ± 0.37$^{\text{d-i}}$	4.90 ± 0.41$^{\text{g-m}}$	68.91 ± 0.41^{lmn}
		50	14.36 ± 1.36$^{\text{c-f}}$	4.66 ± 0.59$^{\text{h-n}}$	83.25 ± 3.25^{f}
		70	16.65 ± 0.53^{ab}	5.76 ± 0.24$^{\text{c-h}}$	66.33 ± 0.56$^{\text{m-p}}$
ChCL-LeA	10	30	9.27 ± 1.87$^{\text{n-s}}$	4.27 ± 1.11$^{\text{j-n}}$	34.07 ± 0.24^{t}
		50	12.53 ± 0.60$^{\text{e-j}}$	6.58 ± 0.07$^{\text{a-f}}$	46.24 ± 0.91^{rs}
		70	15.10 ± 0.20$^{\text{a-d}}$	4.84 ± 0.37$^{\text{g-m}}$	62.22 ± 0.84^{p}
	30	30	11.99 ± 0.05$^{\text{g-k}}$	4.30 ± 0.28$^{\text{i-n}}$	46.90 ± 2.76^{rs}
		50	16.69 ± 1.00^{ab}	6.17 ± 0.17$^{\text{b-g}}$	94.00 ± 0.25^{a}
		70	15.04 ± 0.78$^{\text{a-d}}$	5.16 ± 0.21$^{\text{f-l}}$	76.43 ± 0.23^{hij}
	50	30	14.35 ± 0.70$^{\text{c-f}}$	4.94 ± 0.50$^{\text{g-m}}$	47.47 ± 3.80^{rs}
		50	16.73 ± 1.08^{a}	5.70 ± 0.63$^{\text{c-j}}$	62.24 ± 1.51^{p}
		70	15.46 ± 0.61^{abc}	5.23 ± 0.32$^{\text{e-l}}$	74.17 ± 0.53$^{\text{h-k}}$
Water		30	5.16 ± 0.29$^{\text{w-A}}$	2.30 ± 0.56$^{\text{r-w}}$	66.57 ± 0.31$^{\text{m-p}}$
		50	4.75 ± 0.28$^{\text{x-B}}$	2.61 ± 0.25$^{\text{o-u}}$	68.51 ± 0.42^{lmn}
		70	6.72 ± 0.29$^{\text{t-x}}$	3.81 ± 0.54$^{\text{l-q}}$	75.85 ± 0.31^{hij}
30 % EtOH (*v*/*v*)		30	6.95 ± 0.07$^{\text{t-x}}$	2.74 ± 0.02$^{\text{o-t}}$	85.52 ± 0.00^{def}
		50	9.96 ± 0.01$^{\text{k-q}}$	4.03 ± 0.05$^{\text{k-o}}$	90.45 ± 0.33^{abc}
		70	8.16 ± 0.12$^{\text{p-u}}$	4.90 ± 0.07$^{\text{g-m}}$	72.64 ± 0.39$^{\text{i-l}}$
50 % EtOH (*v*/*v*)		30	6.57 ± 0.35$^{\text{t-y}}$	2.17 ± 0.12$^{\text{s-w}}$	73.90 ± 0.54$^{\text{h-k}}$

	50	$9.30 \pm 0.13^{n\text{-}s}$	$5.84 \pm 0.06^{c\text{-}h}$	$89.70 \pm 0.18^{a\text{-}d}$
	70	$10.45 \pm 0.13^{j\text{-}o}$	7.44 ± 0.03^{ab}	90.86 ± 0.29^{ab}
70 % EtOH (*v*/*v*)	30	$3.09 \pm 0.06^{A\text{-}D}$	$1.10 \pm 0.05^{v\text{-}y}$	43.17 ± 0.62^{s}
	50	$5.78 \pm 0.44^{v\text{-}z}$	$2.65 \pm 0.20^{o\text{-}t}$	$65.57 \pm 0.06^{m\text{-}p}$
	70	$6.67 \pm 0.18^{t\text{-}y}$	$3.94 \pm 0.12^{l\text{-}p}$	$74.44 \pm 0.48^{h\text{-}k}$
MeOH	30	1.18 ± 0.04^{D}	0.33 ± 0.01^{y}	26.68 ± 1.00^{u}
	50	1.56 ± 0.14^{CD}	0.53 ± 0.05^{xy}	33.38 ± 0.06^{t}
	70	1.88 ± 0.16^{CD}	0.38 ± 0.04^{y}	33.79 ± 1.04^{t}

Results are expressed as mean value±standard deviation (N = 3). Different letters within a column indicate a significant difference based on Tukey's HSD test at $p < 0.05$.
